# Application of Microbiome in Forensics

**DOI:** 10.1016/j.gpb.2022.07.007

**Published:** 2022-08-27

**Authors:** Jun Zhang, Wenli Liu, Halimureti Simayijiang, Ping Hu, Jiangwei Yan

**Affiliations:** 1 School of Forensic Medicine, Shanxi Medical University, Taiyuan 030001, China; 2 Beijing Center for Physical and Chemical Analysis, Beijing 100089, China; 3 Key Laboratory of Environment and Health (HUST), Ministry of Education & Ministry of Environmental Protection, and State Key Laboratory of Environmental Health (Incubation), School of Public Health, Tongji Medical College, Huazhong University of Science and Technology, Wuhan 430074, China

**Keywords:** Microbiome, Forensics, Next-generation sequencing, Bioinformatics, Application

## Abstract

Recent advances in **next-generation sequencing** technologies and improvements in **bioinformatics** have expanded the scope of **microbiome** analysis as a **forensic** tool. Microbiome research is concerned with the study of the compositional profile and diversity of microbial flora as well as the interactions between microbes, hosts, and the environment. It has opened up many new possibilities for forensic analysis. In this review, we discuss various **applications** of microbiome in forensics, including identification of individuals, geolocation inference, and post-mortem interval (PMI) estimation.

## Introduction

Microorganisms are microscopic or submicroscopic organisms such as bacteria, fungi, viruses, algae, and some small protozoa [1]. The microbiome consists of the microorganisms and their habitats together with their genomes [2]. In recent years, next-generation sequencing (NGS) technology and bioinformatics have made great strides in expanding our knowledge of microbiomes. Human health, energy production, agriculture, and the environment are all influenced by the microbiome [3]. Numerous large-scale programs have produced massive amounts of data over the past 15 years to characterize microbiomes in niches across the globe, such as the Human Microbiome Project (HMP) [4], [5] and the Earth Microbiome Project (EMP) [6]. The HMP was launched in October 2007 [4] with the aim of exploring the composition and distribution of microbial communities in different regions of the human body and building a database of microbial genome sequences. It also aimed to clarify how microbiology interacts with human health. EMP was launched in August 2010 [6] to build a global database of microbial diversity with a culture-independent approach. Successive large-scale projects have spurred the development of microbiome research into a new era of rapid progress.

The current study on the human microbiome and its environmental influences is also of interest to forensic scientists. This is because each individual has a unique microbial community that differs from that of other individuals, and this particular microbial community can persist over long periods of time [7]. Moreover, different parts of the body also have different microbial communities [8]. For instance, it is commonly believed that the microbial communities in the oral cavity, skin, and gut are more diverse than in other parts of the body [9]. When it comes to the microbiome in the environment, current evidence does not support the hypothesis that “everything is everywhere”, but that microbial communities exhibit a biogeographic pattern. There are marked variations in the structure of microbial communities between different geographic regions [10]. The ubiquity, heterogeneity, and transferability of the environmental microbiome can provide valuable geographic information [11]. There is no doubt that these compelling and interesting findings prove that forensic scientists can analyze microbiomes to solve a variety of problems in forensics.

Indeed, microbes have been used as forensic evidence since the late 19th century [12]. Early applications of microbial forensics focused mainly on the study of the pathogenicity and lethality of microbes. Microbial forensics emerged as a new discipline because letters containing anthrax spores were used as biological weapons in late 2001 [13]. Thereafter, the term microbial forensics was defined as “the discipline of applying scientific methods to the analysis of evidence of a bioterrorism attack, biocrime, hoax, or inadvertent release of a biological agent or toxin with the goal of attribution” [14]. The main objective of this discipline is to detect and identify microorganisms used in biometrics and trace their sources. Such investigations can provide rapid and accurate information on bioterrorism to better predict and prevent related crimes.

Prior to the advent of NGS technology, forensic scientists could not work with microbes because the sequencing techniques required to characterize the microbiota were either too slow and costly or required culture-dependent techniques. With NGS technology, scientists can now accurately, rapidly, and comprehensively determine the DNA sequences of all microorganisms in a sample [15], [16], and avoid experimental contamination and data deviation caused by microbial cultures, which has proven useful in forensics [17]. The original definition of microbial forensics, which focused only on bioterrorism, has simply become too narrow in light of these enormous potential applications in forensics [18]. It is now generally accepted that microbial forensics encompasses “the discipline of characterizing microbiological evidence to develop investigative leads in criminal and civil cases” [14], [19].

Recent developments in microbiome technology have introduced new approaches to forensics. For instance, the microbiome is being used in legal practice to rule out suspects and solve criminal cases [11]. Here, we discuss recent advances in the application of the microbiome in forensics.

## Methods in microbiome research

### Data generation

Amplicon sequencing and shotgun metagenomic sequencing are currently the two main methods for characterizing the microbiome. Amplicon sequencing, which targets marker genes or regions of interest, is the most popular NGS technology for forensic microbiome analysis. Amplicon sequencing of prokaryotic 16S ribosomal RNA (rRNA) genes and eukaryotic internal transcribed spacer (ITS) regions or 18S rRNA genes is the predominant method for profiling microorganisms [20]. It is characterized by high efficiency and low cost through amplification of the 16/18S rRNA genes and the ITS regions. It can be applied to low-biomass specimens without affecting host DNA. The Illumina MiSeq is the most widely used platform for microbiome analysis [21]. Due to the limited sequencing length, this sequencing platform employs region-specific primers, such as the V4 region of 16S rDNA and the V2 region of 18S rDNA. Therefore, this technique has limited resolution at the genus level and is susceptible to the number of polymerase chain reaction (PCR) cycles and primers chosen. In contrast, long-read sequencing platforms, such as Oxford Nanopore Technologies (ONT) and Pacific Biosciences (PacBio), provide solutions for full-length sequencing of hypervariable regions [22], [23]. However, exiting databases need to keep up with the latest technological advances. Therefore, forensic scientists have employed targeted sequencing based on microbial single nucleotide polymorphisms (SNPs) to characterize microbiomes [24]. Shotgun metagenomic datasets have been used to identify SNP markers. In contrast, shotgun metagenomic sequencing provides functional gene information and strain-level resolution, rather than just taxonomic composition as determined by amplicon sequencing [25]. The disadvantage, however, is that the method is more expensive and performs poorly on specimens with low biomass or heavily contaminated by host genomes. Therefore, shotgun metagenomic sequencing is limited in its application to forensics.

### Data analysis

**Figure 1:**
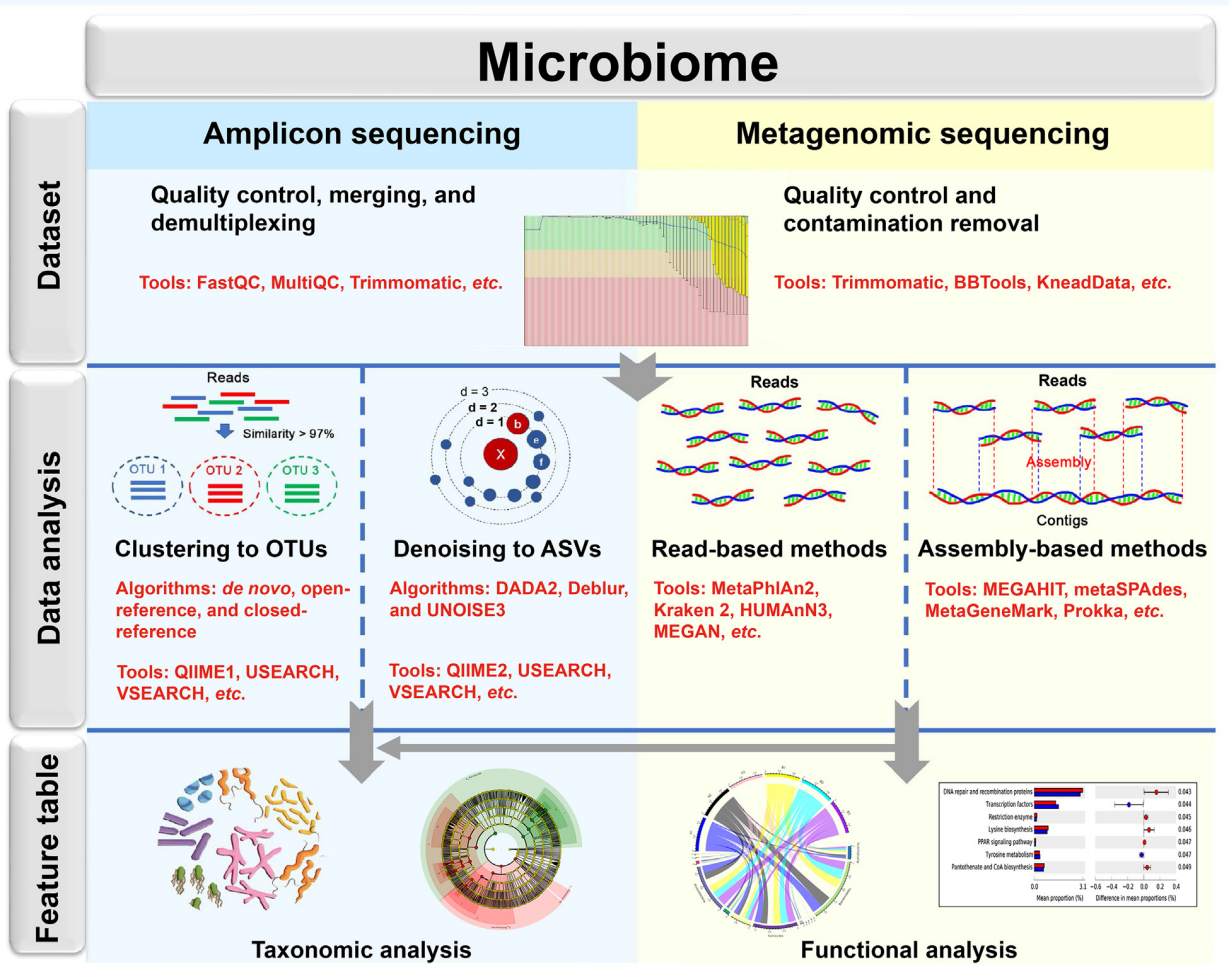
**Graphic summary illustrating the analysis pipelines for amplicon sequencing and shotgun metagenomic sequencing** OTU, operational taxonomic unit; ASV, amplicon sequence variant.

A pipeline for forensic microbiome analysis is still not available. As shown in Figure 1, pipelines comparable with those used in other disciplines are used for forensic microbiome analysis.

In this review, we have discussed the pipelines for amplicon sequencing analysis that are applicable to Illumina sequencing platforms. In amplicon analysis, defining and identifying a unique sequence is challenging. This is because the sequences of hypervariable regions vary widely within the same taxon and even within a single cell. Selecting a sequence that is representative of thousands of sequences in a species has been adopted to solve this problem [2]. Two major categories of methods for selecting representative sequences are clustering into operational taxonomic units (OTUs) and denoising into amplicon sequence variants (ASVs). Clustering of sequences into OTUs is based on a threshold of divergence with 97% or 99% similarity [26]. In general, denoising methods are preferred over clustering methods for amplicon analysis. This is because the ASVs generated by the denoising algorithm are more exactly representative sequences compared to OTUs [27]. Denoising algorithms mainly include DADA2, Deblur, and UNOISE3 [27]. The clustering and denoising algorithms can be performed by several standard amplicon analysis packages, such as Mothur [28], QIIME [29], QIIME2 [30], USEARCH [31], and VSEARCH [32].

The use of representative sequences allows us to analyze amplicon sequencing data without taxonomic information. However, the use of a feature table (OTU/ASV table) without taxonomic information is unfavorable for cross-sectional comparisons of different studies in most cases, because a clustering or denoising process has to be repeated with every addition of new data. Forensic applications or database references are excluded from this mode. The feature table with associated taxonomic information (*e.g.*, kingdom, phylum, class, order, family, genus, and species) is more convenient to use. There are public databases that you can access for taxonomic references, *e.g.*, SILVA [33], Greengenes [34], and UNITE [35].

Shotgun metagenomic sequencing can provide functional information and higher-resolution taxonomic annotation. An increase in the amount of data means that more computational resources and bioinformatics capacity are required. This limits the application of metagenomic sequencing in forensics to some extent. Shotgun metagenomic sequencing can identify all DNA fragments in a sample, including microbial DNA and host DNA [36]. Quality control and removal of contaminants from the raw data are essential steps before taxonomic and functional analyses. The tools KneadData [37], Trimmomatic [38], and BBTools (http://sourceforge.net/projects/bbmap) are commonly used for quality control and host contamination removal. Taxonomic and functional analyses can be performed in two ways: by aligning clean sequences with databases (read-based methods) and by assembling reads into contigs (assembly-based methods) [39]. The tools MetaPhlAn2 [40], Kraken 2 [41], HUMAnN3 [42], and MEGAN [43] are commonly used for taxonomic and functional profiling with the read-based methods. The reads can be assembled into contigs using MEGAHIT [44] or metaSPAdes [45]. Subsequently, the assembled contigs are identified using MetaGeneMark [46] or Prokka [47]. Indeed, metagenomic studies using assembly-based methods are remarkably rare in forensics. Moreover, forensic scientists seem to be more interested in taxonomic information than functional information.

## Application in forensics

**Figure 2:**
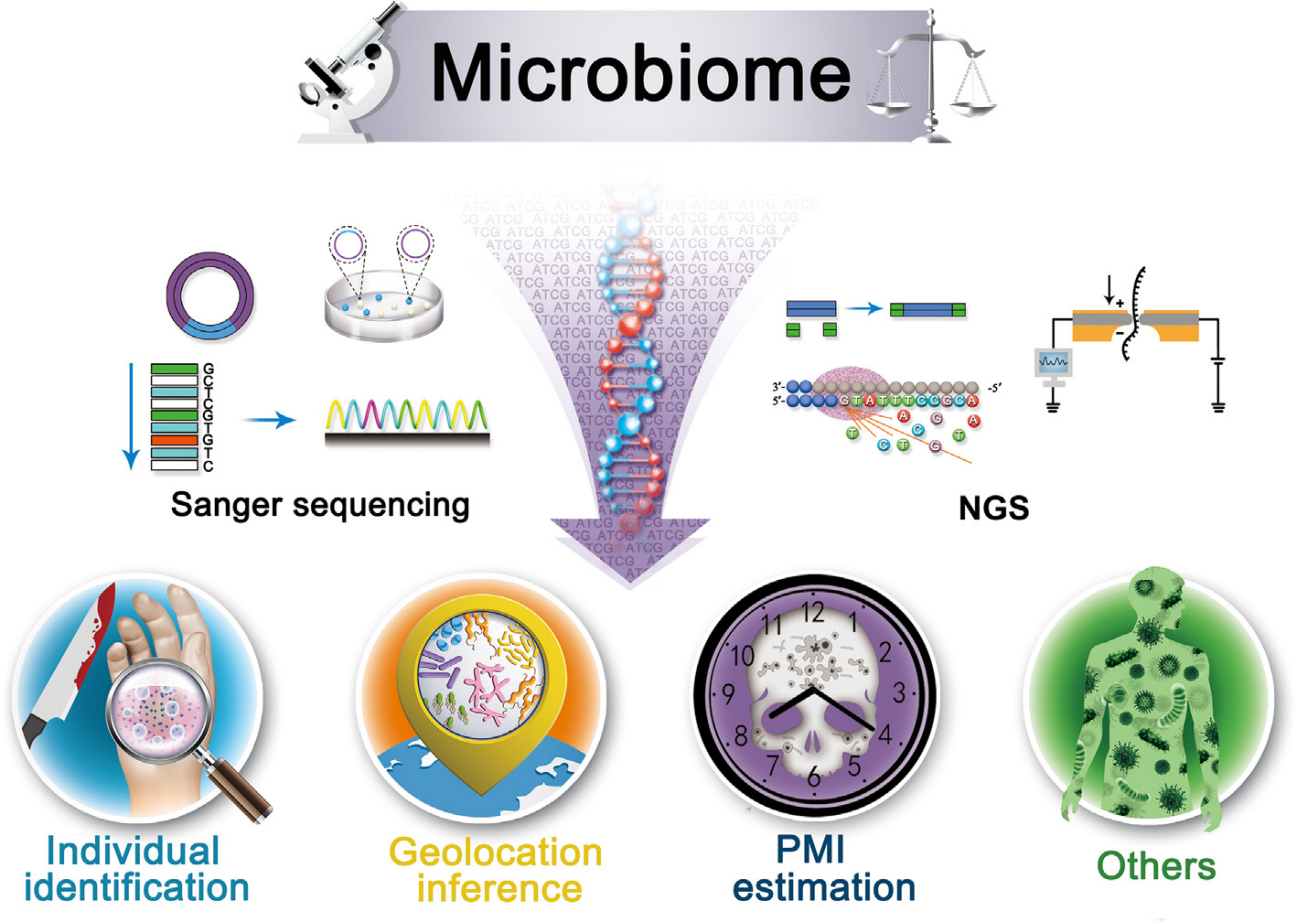
**Forensic analysis by metagenomics** Compared with Sanger sequencing, NGS technology has dramatically reduced the cost of sequencing and increased the throughput and read length, ushering forensic science into a new stage of development. Microbiome may influence many aspects of forensics, including personal identification, geolocation inference, and PMI estimation. In addition to the applications listed, microbiome may facilitate a breakthrough in forensic pathology, toxicology, and tests for substance abuse. Therefore, advances in metagenomics will provide new insights into the field of application forensics. NGS, next-generation sequencing; PMI, post-mortem interval.

A major breakthrough in microbiome research methods, particularly NGS-based technology, has resulted in the increased significance of the microbiome analysis for forensic applications [48], [49]. Analysis of microbiome data, including amplicon and metagenome sequencing data, can aid in many aspects of forensics, including individual identification, geolocation inference, post-mortem interval (PMI) estimation, and more (Figure 2; Table 1).

**Table 1:** **Overview of the application of microbiome in individual identification, geolocation inference, and PMI estimation**

**Application**	**Application foundation**	**Classification**	**Refs.**
Individual identification	Strong variations in community membership between individuals, some of these variations are stable over time	Main tissue origins: skin, oral, gut, and vaginal hair	[7], [55], [57], [59], [62], [63], [64], [66], [67], [68]
		The transfer of microbiomes: cohabitating individuals, direct and indirect transfer, sexual contact, single computer keys and mice, and smartphone	[7], [57], [58], [59], [60]
		New markers: clade-specific markers and CRISPR spacers	[62], [63], [64], [66]
		Influencing factors: decay of microbiota traces with time and diurnal patterns of microbiota	[67], [68]
Geolocation inference	Microbial communities exhibit a biogeographic pattern	Molecular technologies: TRFLP, DGGE, RISA, and NGS (16S rRNA gene, 18S rRNA gene, metabarcoding, and shotgun metagenomics)	[72], [73], [74], [75], [76], [77], [78], [79], [80]
		Sample types: soil, shoe, dust, skin and body fluids, and gut	[60], [72], [73], [74], [75], [76], [77], [78], [79], [81], [82], [83], [84], [85]
PMI estimation	The microbiomes that drive mammalian decomposition are somewhat similar and repeatable across different hosts and environments	Animal models: mice, rat, and swine	[20], [88], [89], [90], [91], [93]
		Human cadaver	[20], [94]
		Sample types: abdomen, skin, scalp, gut, bone, and gravesoil	[20], [88], [89], [90], [91], [93]
		External environments: aboveground, burial cadavers, freshwater, indoor, different seasons, and different sites	[20], [84], [85], [88], [90], [91], [93], [94]

*Note*: PMI, post-mortem interval; CRISPR, clustered regularly interspaced short palindromic repeat; TRFLP, terminal restriction fragment length polymorphism; DGGE, denaturing gel electrophoresis; RISA, ribosomal intergenic spacer analysis; NGS, next-generation sequencing.

### Individual identification

Genotyping of short tandem repeats (STRs) by PCR and capillary electrophoresis (CE) is the mainstay of forensic DNA analysis and is widely used for individual identification and paternity testing [50], [51]. Forensic casework is often hampered by degraded or inadequate DNA samples that make further analysis impossible. However, microorganisms can provide clues that help investigators. According to a recent study, the number of microbial cells in the human body is 1.3 times the total number of human cells [52]. A human microbiota contains more than one million genes, which is about 500 times more than the number of genes in the human genome [53], [54]. In addition, the composition of microbial communities within an individual is influenced by genetic factors, the living environment, and the lifestyle of the host [4]. In theory, each individual carries a unique set of microorganisms that can be identified through microbiome analysis.

Researchers at Harvard University [55] analyzed the microbiomes of saliva, skin, feces, and other body parts of 242 volunteers who participated in the HMP to test the uniqueness and stability of the microbiome in identifying individuals. The result showed that strain-level microbial features associated with humans were sufficient to uniquely identify individuals. A gene-level feature also exhibited more stability over time compared to a taxon-level feature. About 30% of individuals could still be identified after 30–300 days by gene-level analyses of a typical body part, and there were few false-positive matches. The observations from this study suggest that microbiomes in faeces are the most stable (over 80% of individuals could still be clearly identified after one year) and that microbiomes in skin and vaginal areas tend to be more susceptible to interference.

However, microbiomes on body surfaces (*e.g.*, skin and hair) could play a more important role in forensics, as individuals readily transmit their microbiota to other individuals or to the surface of an object when they touch it [56]. Individuals can greatly alter the microbial community in their living environment. Cohabitating individuals had converging skin microbiomes due to direct and frequent contact between individuals and shared surfaces in the household [57]. The transfer of skin microbiomes between individuals who do not cohabit can also be considered a trace evidence [58]. The study by Williams and Gibson [59] showed that it is possible to detect sexual contact using the microbiome from the pubic mound area, as the pubic microbiome can distinguish one individual from another. In women who were sexually assaulted, over 10% of their pubic microbiome was derived from the assailant. If we know who the assailant is, we can determine if sexual contact has occurred. The human microbiome is not only transmitted from person to person, but also persists on touched objects. Researchers have studied the bacteria on people’s hands compared to those on their personal objects, such as computers [7] and smartphones [60]. A relavant correlation was found between the composition of the microbial community on people’s hands and that on the surfaces of their computers and smartphones. Inanimate objects can harbor these bacteria for more than two weeks. Individual activity can also considerably alter the microbial community in their living environment. For instance, the microbiota on a kitchen counter can be matched to the owner shortly after moving into a new house. Each person can contribute to a personal “microbial cloud” by releasing microbes through the air. Most of the individuals can be identified through metagenomic analysis of this “microbial cloud” [61]. Overall, it is possible to identify an individual by analyzing the environmental microbiome.

In the studies which analyze the body surface microbiome for individual identification, the microbiomes are mainly characterized by amplicon sequencing of the 16S rRNA genes. However, this method has limited resolution of species and strains. In a study by Schmedes et al. [62], nucleotide diversity of shotgun metagenomes of skin microbiomes (samples were collected from 12 individuals and 17 skin body sites at three time points over a period of > 2.5 years) was identified as a marker for individual identification. They then introduced hidSkinPlex, a targeted sequencing panel that uses clade-specific markers from skin microbiomes to identify individuals. Skin microbiome profiles generated from the foot, hand, and manubrium could be assigned to the individual host with up to 92%–100% accuracy [62], [63]. They also improved the marker set (hidSkinPlex+), which contains 365 SNPs from 135 markers. The improved marker set is smaller and more robust than the original and can still be used to accurately identify individuals (the Matthews correlation coefficient was 0.949) [64].

Furthermore, the history of bacterial infections can be traced using clustered regularly interspaced short palindromic repeats (CRISPRs) [65]. Targeted sequencing of bacterial CRISPR spacers can provide higher resolution of phylogenetic information than other makers. In the study by Toyomane et al. [66], 24 putative CRISPR arrays were used to characterize individuality by analyzing a shotgun metagenome dataset of human skin. The results showed that CRISPR spacers have high polymorphism in the skin microbiome. CRISPR typing achieved higher accuracy (95.2%) than 16S rRNA gene sequencing (52.6%). However, further studies are needed to characterize the individuality of the body surface microbiome at the gene level.

Identifying individuals from the body surface microbiome is still fraught with challenges in forensics. Wilkins et al. [67] collected skin and household surface microbiota over four seasons to investigate the accuracy of matching individuals to their households over long periods of time. They found that accuracy decreased with the time interval between skin and household surface samples. Most OTUs remained on skin or household surfaces for less than a season. Another study showed that diurnal variation in the human skin microbiome can also reduce the accuracy of identifying individuals from the microbiome [68]. For a total of 160 species, there was a remarkable change in relative abundance between morning and evening at all sampling sites. These results suggest that the temporal decay of microbiota traces and diurnal patterns of microbiota should be considered in the development of the skin microbiome as a potential forensic method for identifying individuals.

### Geolocation inference

As part of a forensic investigation, environmental samples such as soil, water, and even plants can provide valuable clues [69]. A soil investigation can provide vital evidence for identifying suspects and crime scenes, and it can also provide direction and scope for solving cases [70], [71]. Studies on environmental microbiology have shown that there are a variety of microbes that are common in water and soil. Species and strains vary from region to region. Location-specific microbiological information may indicate that a person has recently moved to a new geolocation. With the rapid development of molecular biology and metagenomic technology, our understanding of microbial biogeographical patterns has steadily deepened and the application of microbiome-based inference to location has also been promoted.

Forensic geolocation has already been explored using terminal restriction fragment length polymorphism (TRFLP) [72] and denaturing gel electrophoresis (DGGE) [73]. In recent years, Habtom et al. [74] have used TRFLP to characterize soil microbial DNA profiles at local to regional scales (2 m–260 km) and have found that soil microbial DNA profiles allow conclusions to be drawn about the geographic location of a sample on a scale of at least 25 m.

In microbiology, various molecular techniques were used to identify geolocation, including TRFLP, ribosomal intergenic spacer analysis (RISA), microarrays, and NGS (Roche 454, Ion Torrent, and Illumina MiSeq), as well as n-alkanes and fatty alcohol profiles. The result showed that TRFLP and Illumina MiSeq performed best for 16S rRNA gene sequencing and RISA [75]. Demanèche et al. [76] performed a blinded test to determine whether bacterial communities in soil samples are reliable for forensic analysis. Two biological methods were employed to evaluate the ability to distinguish soil bacterial communities: RISA and 16S rRNA gene sequencing. Both methods were effective in identifying a single soil source. However, characterizing mixed-source soil sample required the combination of both methods. In a study, Yang et al. [77] sequenced the 16S rRNA genes to analyze soil bacterial communities in 529 samples from 61 districts of 10 major cities in China. Random soil samples were assigned to specific districts and cities based on bacterial communities with 66.7% and 90% accuracy, respectively. This research has shown that soil microbes can provide clues to the source of unknown samples and allow comparison of samples taken from suspects or crime scenes.

Most of the publications on geolocation inference have dealt with the bacterial community in soil. Indeed, soil contains a diverse microflora with bacteria, fungi, protozoa, viral flora, and so on. Lilje et al. [78] studied various microbial flora to develop criteria for managing soil metagenomic data and database retrieval for forensic applications. Sequencing data in the study included the 18S rRNA genes of fungi as well as the small subunit ribosomal RNA (SSU rRNA) regions of arbuscular mycorrhizal fungi (AMF), and the 16S rRNA genes of bacteria. For data management, different approaches were used to filter the data. Comparison of the data shows that creating and using a filtered 18S rRNA database would provide much greater computational efficiency and flexibility. Giampaoli et al. [79] used a metabarcoding method to successfully, accurately, and sensitively analyze the biological DNA composition of specific soils, including microflora, plant, metazoan, and protozoan DNA. DNA metabarcoding is a useful method for identifying microbial components in samples that are geologically similar but from different environments. Shotgun metagenomic sequencing allows the simultaneous detection of the entire microflora including bacteria, fungi, viruses, *etc*., in a sample. Therefore, metagenomics is a potential tool for making inferences about geolocation in the context of forensic identification. Danko et al. [80] presented a global atlas of 4758 metagenomic samples collected over three years in 60 cities worldwide. They found that city-specific microbial taxonomic signatures can be used to predict the geolocation of samples with 78.9% accuracy. Based on their findings, successful geolocation of samples based on city-specific taxa could facilitate future forensic biogeographic capacity.

The study by Lax et al. [60] showed that the microbial communities associated with sole from shoes are also associated with the microbiota of the ground on which people walk. Bayesian methodology was used to determine the origin of a shoe sample based on its similarity to a particular ground sample. At a given time point, the composition of the microbial community on the ground had a strong and direct influence on the microbial population living on the corresponding sole. However, the test samples from these studies were generally only compared with several reference samples collected from suspected crime scenes. This limited broader forensic application of geolocation inferences based on the biogeography of the soil microbiome. The establishment of soil microbiome databases and the development of machine learning algorithms could provide available reference databases. Grantham et al. [81] applied the DeepSpace algorithm to a database containing more than 1300 dust microbiome samples in America. The result showed that most geolocation predictions made using this method were less than 100 km from actual locations and that dust-associated fungi alone predicted the location of a sample with nearly 90% accuracy.

In addition to the environmental microbiome, the human-associated microbiome can also provide geographic information. This is because the human-associated microbiome is partly influenced by many factors such as diet, geographic factors, and the degree of urbanization [82]. These specific microbiomes may contain geographic information about the host. He et al. [83] characterized the gut microbial communities of 7009 individuals from 14 districts within one province in China and found that host location was most strongly associated with microbial community variation. The proportion of Firmicutes and Bacteroidetes in the gut microbiota differed with latitude. This could allow conclusions to be drawn about whether a person is from the northern or southern hemisphere [84]. Singh et al. [85] used the Forensic Microbiome Database (FMD), a database of microbiome data from human skin, vaginal fluid, saliva, and stool from 35 countries, and they found that the distribution of the human microbiome varied depending on the geographical location of the host.

### PMI estimation

PMI estimation has always been an important tool in the fight against crime. Traditional physical, histomorphological, and biochemical techniques have been used to estimate PMI. However, traditional methods are compromised by the preservation of the material and the time limit of the degradation of endogenous substances. Beyond this time limit, accuracy decreases sharply. Metagenomics could provide another solution to this problem. After death, the cadaver gradually decomposes under the action of microorganisms. In the meantime, spoilage products also gradually accumulate in the tissue. The amount of spoilage microorganisms and spoilage products changes according to certain patterns as the prolongation of the death time. Therefore, PMI estimation can be derived from the law of microbial community succession. Recently, metagenomic analysis has become increasingly important in PMI estimation [86].

The microbiomes that drive mammalian decomposition are somewhat similar and repeatable in different hosts and environments [87]. This ecological hypothesis underpins the microbiome-based method of PMI estimation. Most tightly controlled experiments have been performed on animal models rather than human bodies. Metcalf et al. [88] collected samples of abdomen, body skin, scalp, and soil in a mouse cadaver decomposition system to characterize the bacterial and microbial eukaryotic ecology associated with cadaver decomposition. Their study provided a PMI estimate with a mean absolute error of about 3 days over 48 days. The lowest error was obtained with scalp data. The study suggests that cadaver microbiome data can be considered as a potential forensic tool for PMI estimation.

Subsequently, further research has been published on the cadaver microbiome in different animals, on different body sites, and in different environments. Guo et al. [89] investigated the bacterial communities that were degraded in the remains of rats under natural conditions or conditions that excluded pests. The relative abundance of the dominant phyla (Proteobacteria, Firmicutes, Bacteroidetes, and Actinobacteria) changed significantly during disintegration of the body; the predominant bacterial type shifted from aerobic to anaerobic and the community composition became more similar between body parts. In forensics, cadavers can also be buried in soil and submerged in water. Zhang et al. [90] characterized the bacterial communities from the soil, cecum, and skin of buried cadavers during decomposition to predict PMI in a SD rat model system. The prediction model yielded a mean absolute error of 1.82, 2.06, and 2.13 days, respectively, within 60 days of decomposition. Cartozzo et al. [91] predicted the post-mortem submersion interval (PMSI) of porcine skeletal remains in a freshwater lake based on the microbiome and obtained a mean absolute error of 37–57 days within 579 days of the experiment. Most studies estimating PMI based on the microbiome of the cadavers have used random forest algorithm to determine the predicted value. The study by Liu et al. [92] showed that an artificial neural network (ANN) model could improve the prediction accuracy of PMI, so that PMI could be predicted within about 1.5 h over 24 h and 14.5 h over 15 days of decomposition. This research suggests that cadaveric microbial communities change in predictable successional processes.

Microbial variations in cadavers are closely related to external environmental factors. Carter et al. [93] studied the succession of microbial communities in soil under porcine cadavers in summer and winter, respectively. The authors found that the season of decomposition had a considerable effect on the composition of microbial communities (including bacteria, archaea, and eukaryotes). Metcalf et al. [20] studied the microbiome of mice cadaver in soil from three different sites (desert, short grass, and forest) based on 16S rRNA, 18S rRNA, and ITS gene sequencing methods. The result confirmed that microbial succession was predictable regardless of soil type, seasons, or the presence of other scavengers.

However, it is important to include human cadavers in the study for the method to be applicable in forensics. Metcalf et al. [20] observed reproducible succession of microbiota within the season and accurate PMI estimation in the experiment with human cadavers. Furthermore, the study by DeBruyn and Hauther [94] showed that changes in human microbial communities can become forensic tools for PMI estimation. The researchers continuously collected samples from the cecum of a cadaver during the bloat stage of decomposition (under natural conditions), and then determined the pattern of succession in the intestinal bacterial community after human death by sequencing the 16S rRNA gene amplicon. The sequencing results showed that the abundance of Bacteroidales decreased over time. In contrast, the abundance of Clostridiales and fly-associated Gammaproteobacteria increased. The absolute abundance of bacteria increased significantly, whereas bacterial alpha diversity decreased. The application of the method in forensic cases is limited mainly by the lack of a predictive model based on a sufficient number of human cadaver samples. However, there is enormous potential for developing cadaveric microbiomes as a “clock” for estimating human PMI.

### Other applications

The human-associated microbiome could provide various clues to solve crimes, such as the origin of tissue samples, the time since body fluids were deposited, ethnicity, and possible living conditions. Different organisms or body fluids carry different types of bacteria that can be identified in this way. For instance, vaginal discharge often contains *Lactobacillus crispatus*, *Lactobacillus jensenii*, and *Atopobium vaginae* [95], [96]. Saliva often contains *Streptococcus salivarius* and *Streptococcus mutans* [97], [98]. Hanssen et al.  [99] used saliva deposited on the skin as a study model using 16S rRNA gene sequencing to prove the recognition ability of the human-associated microbiome for body fluid prediction. The microbial composition features can be used to distinguish saliva from skin, and the cross-validation accuracy was 94%. López et al. [100] collected 16S rRNA gene sequencing data from the HMP of 1636 skin, vaginal, and oral samples and then trained 50 taxonomy-independent deep learning networks to classify the origin of the tissue. The result showed that the accuracy of tissue origin classification was very high. The area under curve (AUC) values for skin reached 0.99, for oral secretions reached 0.99, and for vaginal secretion reached 1.00. Furthermore, Dobay et al. [101] collected fluid/tissue samples from six different human body sites. They then exposed these samples to indoor conditions. These samples could still be accurately matched to the corresponding body sites after 30 days of exposure. These results illustrate the potential of microbial diversity profiling as a new forensic tool for identifying body fluids.

Similar to PMI estimation, the microbiome can also provide an available method for estimating the deposition time of body fluid stains. López et al. [102] first use the microbiome of human salivary stains to confirm the feasibility of the method. They identified 15 abundant species using publicly available 16S rRNA gene sequencing data from 1848 saliva samples. They also characterized salivary stains from two individuals exposed to indoor conditions over one year. *Fusobacterium periodonticum*, *Haemophilus parainfluenzae*, *Veillonella dispar*, and *Veillonella parvula* were selected as those whose abundance showed significant time-dependent changes. They then analyzed the salivary stains of 15 individuals exposed to indoor conditions for up to 1 month using the aforementioned markers. The mean absolute error ranged from 3.3 to 7.8 days. To date, there are few studies that estimate the deposition time of other stains in body fluids other than saliva.

Metagenomic analysis of microbial communities could also provide information about an individual’s race and ethnicity. For instance, Yanomomi natives in Venezuela have never had contact with Westerners and have never been exposed to commercial antibiotics; the bacterial diversity and function of their skin is twice that of Americans [103]. However, differences in the human microbiome between ethnic groups are partly mediated by diet, lifestyle, and geographic environment. Deschasaux et al.  [104] showed that individuals of different ethnicities living in the same city tended to have similar gut microbiota characteristics. Borrello et al. [105] found that overall diet quality and dietary intake components can significantly explain ethnic variations in gut bacterial composition. Moreover, body site is a more important determinant factor than ethnic diversity in the human skin microbiota [106]. These findings suggest that classification by race and ethnicity based on the human-associated microbiome should be used with caution.

## Challenges and perspectives

The application of metagenomics in forensics is just beginning. As far as we know, microbiomes are not yet approved as evidence for individual identification, geolocation inference, and PMI estimation. First, there are not yet any standardized operating principles and specifications for the extraction, packaging, transport, and preservation of microbial evidence. Second, the reliability of microbiome tools for forensics needs to be improved. Compared to human DNA markers, the specificity and stability of individual microbes need to be further validated. The reliability of the microbiome for forensic applications needs to be better studied, and reliable error rates need to be established. The problem should be addressed by sufficiently large sample size and quantitative machine learning methods. There are well-tested algorithms for both classification and regression that have been applied in forensics based on the microbiome, including (but not limited to) K-nearest neighbors models [104], random forest model [90], [107], and neural networks [81], [92], [108]. Machine learning methods showed an obvious advantage in managing multidimensional data such as microbiome data [109]. However, quantitative computation of relevant forensic parameters is required. The current evaluation criteria for explaining microbiome evidence differ from the traditional likelihood calculation for human DNA. The evaluation criteria need further research to be accepted by the forensic science community. On the other hand, a sufficient sample size is required for machine learning methods to perform adequately. Therefore, the establishment of microbiome databases is necessary for the application of the method. The establishment of forensic DNA databases has helped the police to identify or exclude persons associated with a crime. It has also enabled the identification of serial offenders by linking multiple cases, which has greatly improved the evidential value of forensic data. Although various microbiome databases such as HMP and EMP have been launched successively, the fragmented state of the publicly available databases has limited the application of microbiome as a forensic tool. Singh et al. [85] reported that they introduced the FMD by collecting 16S rRNA gene sequencing data from publicly available databases to make inferences about the site of discovery. More databases are needed for various forensic purposes. Finally, creating awareness is an essential step in making forensic science permissible. Training and equipment for sequencing microbiome cost a lot. Advances in sequencing technology and computer power will reduce costs. Overall, these issues are critical to the forensic practice of microbiomes and will be overcome as research progresses.

Beyond the applications listed here, metagenomics could bring breakthroughs in forensic pathology, toxicology, and drug abuse testing. In short, the development of metagenomics in forensics will provide a new perspective and new solutions for forensic identification.

## Competing interests

The authors have declared no competing interests.

## CRediT authorship contribution statement

**Jun Zhang:** Writing – original draft, Writing – review & editing, Visualization, Funding acquisition. **Wenli Liu:** Writing – original draft, Visualization. **Halimureti Simayijiang:** Writing – original draft, Writing – review & editing. **Ping Hu:** Conceptualization, Writing – review & editing. **Jiangwei Yan:** Conceptualization, Writing – original draft, Writing – review & editing, Funding acquisition. All authors have read and approved the final manuscript.
